# The role of perceptions and knowledge of leprosy in the elimination of leprosy: A baseline study in Fatehpur district, northern India

**DOI:** 10.1371/journal.pntd.0007302

**Published:** 2019-04-05

**Authors:** Anna T. van ‘t Noordende, Ida J. Korfage, Suchitra Lisam, Mohammed A. Arif, Anil Kumar, Wim H. van Brakel

**Affiliations:** 1 NLR, Amsterdam, Netherlands; 2 Erasmus University Medical Center Rotterdam, Rotterdam, Netherlands; 3 Disability Studies in the Netherlands, Amsterdam, Netherlands; 4 NLR India, New Delhi, India; 5 Central Leprosy Division, Ministry of Health and Family Welfare, New Delhi, India; Lowell General Hospital, UNITED STATES

## Abstract

**Background:**

With the introduction of new interventions to prevent leprosy, such as post-exposure prophylaxis (PEP) given to contacts of leprosy patients, it is necessary to update our understanding of knowledge and perception of leprosy among the populations where these interventions will be introduced, in order to tailor communication optimally to the current situation. This study is a baseline study of the PEP++ project and aimed to assess the knowledge, attitudes and practices regarding leprosy in Fatehpur, India.

**Methodology:**

The study used a community-based cross-sectional design with a mixed-methods approach. We assessed knowledge, attitudes, and practices with the KAP measure, and stigma with the Explanatory Model Interview Catalogue community stigma scale (EMIC-CSS) and the Social Distance Scale (SDS). In addition, semi-structured interviews and focus group discussions were conducted with all participant groups. The quantitative data were analysed using stepwise multivariate regression. The qualitative data were analysed using open, inductive coding and content analysis.

**Findings:**

A total of 446 participants were included in the study: 100 persons affected by leprosy, 111 close contacts, 185 community members and 50 health care workers. In addition, 24 in-depth interviews were conducted and 35 people were included in focus group discussions. 12.5% of the participants had adequate knowledge of leprosy, while 22% had poor knowledge. Knowledge on cause (answered correctly by 10% of the participants), mode of transmission (5%) and symptoms of leprosy (16%) was especially poor. The mean EMIC-CSS score was 15.3 (95%CI 14.6–16.0) and mean SDS score 7.2 (95%CI 6.6–7.8). Better knowledge of leprosy was associated with lower levels of social distance towards persons affected by leprosy.

**Conclusion:**

This study revealed poor knowledge regarding leprosy and high levels of stigma and fear and desire to keep social distance towards persons affected by leprosy. Community education that takes cultural beliefs, knowledge gaps and fears into consideration could improve knowledge, reduce misconceptions and positively influence the perception of leprosy.

## Author summary

With the introduction of new interventions to prevent leprosy, such as post-exposure prophylaxis (PEP) given to contacts of leprosy patients, it is necessary to update our understanding of knowledge and perception of leprosy among the populations where these interventions will be introduced, in order to tailor communication optimally to the current situation. This study aimed to assess the knowledge, attitudes and practices regarding leprosy in Fatehpur, Uttar Pradesh, India. A cross-sectional study design with a mixed-methods approach was used. We assessed knowledge, attitudes, and practices with the KAP measure, and stigma with the Explanatory Model Interview Catalogue community stigma scale (EMIC-CSS) and the Social Distance Scale (SDS). A total of 446 participants were included: 100 people affected by leprosy, 111 close contacts, 185 community members and 50 health care workers. In addition, 24 in-depth interviews were conducted and 35 people were included in focus group discussions. This study revealed poor knowledge regarding leprosy among all four participant groups. Knowledge on mode of transmission, cause and symptoms of leprosy was especially poor. In addition, we found high levels of stigma and social distance towards people affected by leprosy. Community education that takes cultural beliefs, knowledge gaps and fears into consideration could improve knowledge, reduce misconceptions and positively influence the perception of leprosy.

## Introduction

Leprosy is an infectious disease caused by *Mycobacterium leprae*. Leprosy primarily affects the peripheral nerves and skin. The damage of the nerves may affect the sensory, motor and autonomic functions of the nerves, resulting ultimately in disability [1,2]. In addition to the physical consequences of leprosy, social stigmatization is a challenge for many persons affected by leprosy, especially since this often remains once the medical treatment is finished [3–5].Transmission of the bacteria is believed to occur through long‐term exposure of the respiratory system to airborne nasal droplets [1,2,6].

With over 210,000 new patients diagnosed in the world each year, leprosy is still a public health problem in many low and middle income countries [2,7]. To interrupt the transmission of *M*. *leprae* and to reduce the number of new leprosy patients globally early detection and prompt treatment with multi-drug therapy (MDT) are essential [2,8,9]. Early detection is also necessary to reduce the physical and social consequences of the disease as the complications of leprosy depend on how timely in the disease process leprosy is diagnosed and treated [8,10]. Prevention of disability thus begins with early detection of leprosy [8].

Late detection of leprosy is associated with misdiagnosis, inadequate or incorrect knowledge about the disease as well as negative beliefs about leprosy among persons affected and health care workers [10]. People’s perception of leprosy influences their awareness that certain signs and symptoms may be due to leprosy [10,11]. Indeed, voluntary and early reporting of leprosy requires awareness of leprosy and its treatment facilities [12]. Several studies attributed delayed diagnosis of leprosy to the use of traditional medicine and/or low awareness of modern treatment, ignorance of leprosy, a belief in self-cure, unavailability of services or skilled health care workers, stigma and the influence of traditional or community leaders [10,13–17]. In addition, because of the stigma associated with leprosy, persons affected by leprosy often delay seeking treatment until they develop permanent, visible disabilities [2]. This set of interrelated factors suggests that sufficient knowledge of leprosy presentation, clinical features and services and stigma reduction are essential for early detection of leprosy.

Improving the knowledge and perception of leprosy and reducing stigma seem essential to improve strategies for early case detection in leprosy. There have been several studies already that looked into the knowledge, attitudes and practices regarding leprosy of persons affected, the general community, students as well as health care workers. In India alone, over 14 studies that looked into this have been conducted after the year 2000 [12,18–30]. Most of these studies found low levels of knowledge about leprosy and negative attitudes towards persons affected by leprosy. However, most studies administered questionnaires only: only two of these studies conducted in-depth interviews [27,30] and one study used a mixed-methods approach [26]. With the introduction of new interventions to prevent leprosy, such as post-exposure prophylaxis (PEP) given to contacts of leprosy patients, it is necessary to update our understanding of knowledge and perception of leprosy among the populations where these interventions will be introduced, in order to tailor communication optimally to the current situation.

The current study aimed to assess the knowledge, attitudes and practices regarding leprosy in a leprosy endemic district (Fatehpur district in Uttar Pradesh, India) using a mixed-methods approach. A mixed-methods approach will allow us to quantify the knowledge levels as well as gain more insight into the rationale behind people’s perceptions, beliefs and attitudes. We expect that the findings of this study will give insight in the existing knowledge of leprosy, myths or misconceptions, as well as the prevailing attitudes, beliefs and specific fears and concerns people may have about leprosy.

## Methods

### Study design

The study used a community-based cross-sectional design. A mixed methods approach was used. Quantitative questionnaire interviews were used to assess the knowledge and attitudes of people towards leprosy and both semi-structured interviews and focus group discussions were used to ask in-depth questions.

### Study site

The study was conducted in Fatehpur district, Uttar Pradesh, northern India between December 2017 and February 2018. Fatehpur is a district in Uttar Pradesh, a state in northern India, where the prevalence of leprosy is high compared to the national average. The prevalence of leprosy in India, that accounts for more than half (60%) of the global disease burden of leprosy, was 0.69 per 10,000 population in April 2015, the prevalence in Fatehpur was 0.77 per 10,000 population [31,32] indicating the need to put in extra efforts.

### Study population and sample

Four groups of people were included in the study: (1) persons affected by leprosy or “index patients”; (2) close contacts of index patients; (3) community members; and (4) health care workers.

We collected quantitative and qualitative data. Epi Info StatCalc for cross-sectional studies was used to calculate the quantitative sample size. For the quantitative questionnaire interviews we aimed to include a random sample of at least 100 persons of each target group. This estimate is based on an assumed prevalence of ‘negative attitudes’ of 50% at baseline and wanting to be able to detect a reduction of 20% or more (i.e. prevalence of negative attitudes in the 2nd survey is 30% or less). Using these parameters, a significance level of 0.05 and a power of 80%, at least 186 subjects are needed in each group, 93 before and 93 after the community education intervention that will be implemented at a later stage as part of the larger research project (PEP++ project). In addition, we conducted interviews to gain more insight into the rationale behind people’s perceptions, beliefs and attitudes. We aimed to have semi-structured interviews with six persons from each participant group. We also aimed to conduct one focus group discussion per participant group with seven to ten participants each. The participants in the qualitative sample will be a subset of those in the quantitative sample.

### Eligibility criteria

Participants had to live in Fatehpur district. Index patients had to be diagnosed with leprosy within the last five years. Closest contacts included household contacts, family members, neighbours and/or social contacts who have intensive contact with the index patient (at least 20 hours per week for at least three months in the year before the index patient was diagnosed). Community members had to live in the same village or neighbourhood as the index patient. Health care workers had to work in the primary health care centre in the district.

Persons below the age of 16 and persons unwilling or unable to give informed consent were excluded. Close contacts, community members and health care workers were also excluded if they were or had ever been affected by leprosy. Participants who were listed as close contact of an index patient could not participate as community member also.

### Sampling methods

The villages were selected by stratified systematic sampling with a random start from among the villages where one or more index patient lived. A list of 13 blocks and 242 villages in these blocks where new leprosy cases were reported in the year 2016 and 2017 was prepared. Every second village from the total number of villages in each block was selected. The first index patient and village that were visited were selected randomly from the list. A total of 114 villages, spread across the 13 blocks, connected to all the 17 primary health care centres in the district were included.

Participants for the quantitative questionnaire interviews were selected as follows:

The index patients included in the study were selected by stratified systematic sampling with a random start from a list of leprosy patients registered at the primary health care centre.

The close contacts of index patients, i.e. household members, neighbours or social contacts, were selected by the index patient because of their convenient accessibility and proximity. Close contacts were selected by convenience sampling. One contact per index patient was included in the quantitative questionnaire interviews.

The community participants were selected by convenience sampling from among those living in the same village or neighbourhood as an index patient. One or two community members per index patient were selected from within a radius of 500 meters of the house of the index patient in the villages where the index patients live. We aimed to select the community members as randomly as possible while trying to ensure an equal number of men and women.

Health care workers were selected based on convenience sampling. All primary health care centres in the district were visited, where health care workers were selected based on their availability. We included different types of health care workers: auxiliary nurse midwives, non-medical supervisors and assistants, physiotherapists, paramedical workers, medical workers and district leprosy consultants. Half of the health care workers (n = 25) included in this study had specific responsibilities for leprosy treatment services.

The participants for the qualitative interviews were selected using purposive sampling to ensure adequate representation of age, sex and villages. These participants were a subset of those in the quantitative sample. We used a sampling grid to ensure an equal number of men and women were included.

### Data collection

Demographic information was obtained from each participant. In addition, three instruments were used in this study. A knowledge attitudes and practices (KAP) measure and two short stigma instruments: the Explanatory Model Interview Catalogue community stigma scale (EMIC-CSS) and the Social Distance Scale (SDS). All measures were interviewer-administered.

The KAP measure was developed to assess the knowledge, attitudes and perceived practices of index patients, contacts, community members and health care workers regarding leprosy. The KAP measure has been used in several leprosy studies between 2012 and 2017 but the results of these studies have not been published. The questionnaire has 17 items (and consists of single and multiple answer questions). Participants could give multiple answers to some of the KAP questions. Answer options were not suggested to respondents in advance. For the questions for which multiple answers could be given, we considered an answer correct only if the correct answer was given in the absence of incorrect answers. We defined adequate knowledge as 70% or more correct answers on the knowledge section of the KAP measure (≥5 out of 7 questions) Poor knowledge was defined as 30% or less correct answers (≤2 out of 7).

The 15-item EMIC-CSS was used to measure perceived attitudes and behaviour towards persons affected by leprosy. The EMIC-CSS has been validated among community members of persons affected by leprosy in India [33]. The 7-item SDS was used to assess the social distance the interviewee wants to keep towards persons affected by leprosy as a proxy for their attitudes. The SDS has not been formally validated for use with persons affected by leprosy in India, but has been validated among community members of persons affected by leprosy in Indonesia [34]. The SDS has been translated to Hindi, partially validated and used in a study in Uttar Pradesh, India (Ballering et al., in preparation). The EMIC-CSS and SDS were interviewer-administered to community members, close contacts and health care workers.

In addition, cross-sectional data on attitudes and perceptions of the participants towards leprosy were obtained using semi-structured in-depth interviews and focus group discussions.

All interviewers were trained in leprosy, in the instruments used and in the interviewing techniques prior to data collection. Pilot interviews were conducted prior to the final data collection and minor revisions to the interview guide were made. These participants were not included in the final sample and no changes were made to the questionnaires used. All participants were interviewed in their local language by a local interviewer in their home, or at a private space near their home. The in-depth interviews and focus group discussions were audio recorded. A district coordinator monitored the entire process.

### Data analysis

Quantitative data were entered in a database created using Epi Info. Simple descriptive methods were used to generate a demographic profile of the study sample. In addition, mean total scores of the KAP, EMIC-CSS and SDS measures were calculated per participant group. Multivariate regression was done to examine which factors had an independent effect on the outcomes (knowledge, attitudes and perceived stigma). We used stepwise multivariate regression with backward elimination to see if there were associations between knowledge, stigma and social distance and the other variables in our dataset (e.g. gender, occupation, etc.). The multivariate analysis was carried out using a model with all variables potentially associated with the outcome with a *p*-value of <0.2 identified through univariate analysis. Variables with *p*-values of ≥0.05 were eliminated one-by-one until all variables that remained in the model were statistically significant (*p*<0.05). For dependent variables that were distributed non-normally we conducted bootstrapped stepwise multivariate regression with backward elimination, as bootstrapping corrects for non-normality by making no assumptions about the shape of the distribution. Data analysis was done in the software packages Epi Info version 7.2.2.2 and SPSS Statistics version 24.

The recordings of the in-depth interviews and focus group discussions were transcribed, translated to English and analysed using open, inductive coding and content analysis. Similar phrases with recurring themes were coded in the software programme Nvivo version 12 and clustered together in tables, to identify connections.

### Ethical considerations

Ethical approval was obtained from the Institutional Ethics Committee. Ethical approval for this study was obtained as part of a larger research project: the Post-Exposure Prophylaxis project (PEP++ project). All participants were fully informed about the nature and objective of the study and of confidentiality of the data prior to data collection. Written consent for participation in the study was obtained from each participant in their local language. All persons approached agreed to participate in the study.

## Results

### Demographic information

A total of 446 participants, of which 285 men (64%) were included in the study. Four groups of people were included in the study: 100 persons affected by leprosy or “index patients” (22%), 111 close contacts of index patients (25%), 185 community members (41%) and 50 health care workers (11%). Most participants (n = 395, 88%) lived in rural areas. The average age was 39.2 (range 16–90), men were on average older (41.2) than women (35.7). One fifth of the participants were illiterate (n = 95, 21%) and almost one tenth could read and write, but did not have any formal education (n = 37, 8%). Four hundred six participants were Hindu (91%). Over half (n = 225, 65%) of the participants, excluding index patients, had a close relationship with someone with leprosy. Index patients were diagnosed on average 17.9 months ago (range 9–60 months). Half of the health care workers (n = 25, 50%) who were included in this study had specific responsibilities for leprosy treatment services.

Twenty-four in-depth interviews were conducted to supplement the quantitative data. Six people from all four groups (index patients, close contacts, community members and health care workers) were interviewed. Half of them were men (n = 12, 50%). The average age of the interviewees was 31 years for women (range 15–55) and 36 years for men (range 20–57). In addition, 35 people were included in focus group discussions (Table 1). Health care workers and community members were the main sources for participants to acquire leprosy relation information. All health care workers who were interviewed in-depth received training on leprosy.

**Table 1:** Number of participants included in the study, per participant group.

Participant type	Questionnaires ^a^	In-depth interviews	Focus group discussion
Index patient	100	6	9
Close contact	111	6	10
Community member	185	6	7
Health care worker	50	6	9
Total	446	24 ^b^	35^b^

^a^ Index patients were administered the KAP only, while the other participant groups received the KAP, SDS and EMIC-CSS.

^b^ The qualitative respondents are a subset of those in the quantitative sample.

### Knowledge of leprosy

Table 2 provides an overview of the responses given to the KAP measure.

**Table 2:** An overview of the responses given per knowledge question. The responses in green are the correct answers.

Topic	Responses given as percentage of participants who gave the answer as *n* (%).					Percentage of people who gave the correct answer only^a^ (n = 446)
		Persons affected (n = 100)	Contacts (n = 111)	Community (n = 185)	Health workers (n = 50)	
*Early symptoms*	Skin patches	62 (62)	49 (44)	83 (45)	34 (68)	16%
	Loss of sensation	54 (54)	20 (18)	25 (14)	35 (70)	
	Don’t know	10 (10)	34 (31)	57 (31)	4 (8)	
	Itchiness	17 (17)	23 (21)	48 (26)	6 (12)	
	Other: tingling, coughing, bleeding, blisters, rashes	19 (19)	20 (18)	22 (12)	14 (28)	
*Cause of leprosy*	Don’t know	82 (82)	83 (75)	119 (64)	14 (28)	
	Germs/bacteria	6 (6)	11 (10)	15 (8)	25 (50)	10%
	Unclean environment	5 (5)	9 (8)	21 (11)	5 (10)	
	Other: punishments for sins, karma, impure blood, hereditary	10 (10)	11 (10)	32 (17)	5 (10)	
*Transmission of leprosy*	Don’t know	65 (65)	65 (59)	84 (45)	13 (26)	
	Skin contact	23 (23)	33 (30)	71 (38)	16 (32)	
	Eating together	11 (11)	13 (12)	17 (9)	4 (8)	
	Other: contaminated soil, insects, ‘different’	19 (19)	12 (11)	6 (3)	5 (10)	
	By air	4 (4)	3 (3)	7 (4)	8 (16)	2%
*Treatability of leprosy*	Can be treated	97 (97)	102 (93)	168 (91)	49 (98)	93%
	Don’t know	1 (1)	7 (6)	12 (6)	1 (2)	
	Can’t be treated	2 (2)	1 (1)	5 (3)	0 (0)	
*Treated how*	By medication	96 (96)	105 (95)	162 (88)	49 (98)	97%
	Other	2 (2)	1 (1)	10 (5)	1 (1)	
*Contagiousness*	Not contagious when on treatment	45 (45)	56 (50)	81 (44)	26 (52)	54%
	Contagious when on treatment	35 (35)	33 (30)	56 (30)	20 (40)	
	Don’t know	17 (17)	15 (14)	31 (17)	3 (6)	
*Disabilities*	Disabilities can be prevented	61 (61)	72 (65)	117 (63)	41 (82)	65%
	Don’t know	22 (22)	28 (25)	43 (23)	2 (4)	
	Disabilities can’t be prevented	17 (17)	11 (10)	25 (14)	7 (14)	
*Duration of condition*	Leprosy is temporary	50 (50)	44 (40)	92 (50)	26 (52)	48%
	Leprosy is permanent	28 (28)	37 (33)	35 (19)	17 (34)	
	Don’t know	22 (22)	30 (27)	58 (31)	7 (14)	

^a^ In the absence of incorrect answers. Participants could give multiple answers to some of the KAP questions. We choose to present the answers as percentage of participants who gave the answer, rather than as percentage of all the responses given to a particular question. Therefore, the percentages presented may exceed 100%.

Seventy participants (16%) correctly answered that both “loss of sensation” and “skin patches” are early symptoms of leprosy. One community member described the early symptoms of leprosy in one of the interviews as:

*“…Hand or some body parts get numb*, *or they do not know if a needle is pricked and do not realize that a needle has been kept there*. *Malformed fingers*, *water discharge*, *melting of nails*, *the body becomes bowed…” (Community member*, *male*, *42)*

When asked what participants thought was the cause of leprosy, two-thirds (67%, n = 298) indicated they didn’t know. Few participants (n = 43, 10%) only gave the correct answer, “germs or bacteria”, in the absence of wrong answers. A lack of hygiene or cleanliness and eating bad food were often mentioned as causes of leprosy during the in-depth interviews.

Participants were also asked how they thought leprosy is transmitted. There were 11 participants (2%) who only responded that leprosy is transmitted by air. Many of the people who were interviewed in-depth said that they or people from their communities thought that leprosy is transmitted by touch.

A community member described the transmission of leprosy as:

*“…It can happen because of uncleanliness*, *by insects*, *it can spread through touch and by clothes also*. *(…) Leprosy is a disease which can spread even by touch and it casts effect on the people who live with a leprosy patient*. *We should stay away from leprosy patient otherwise it can happen to others also…” (Community member*, *male*, *28)*

A health care worker explained:

*“…Society says not to touch leprosy patients*. *Then we make them understand and show them by touching patients that it is not a disease that spreads by touch…” (Health care worker*, *female*, *55)*

The majority of participants (n = 416, 93%) were aware that leprosy can be treated. Almost all participants (n = 412, 97%) who knew leprosy can be treated knew it can be treated by medication. Over half of the participants (n = 208, 54%) were aware that leprosy is no longer contagious after a patient has started treatment. Two-thirds of the participants (n = 291, 65%) said that the disabilities that some patients have can be prevented, which is correct. Furthermore, when asked if participants thought leprosy was more likely to be temporary or permanent, half (n = 212, 48%) of the participants indicated that they thought leprosy was temporary, which is correct.

### Adequate knowledge of leprosy

Participants answered on average 3.2 out of the 7 KAP questions correctly (range 0–7). Two participants answered all seven questions correctly. With a mean of 4.2 correct answers, health care workers had significantly better knowledge scores (*p* = 0.042) than the other participants (mean knowledge score 3.1). An overview of the number of correct answers per KAP question can be found in Figure A in S1 Fig.

One in eight participants (n = 56, 12.5%) were considered to have adequate knowledge of leprosy, while almost one quarter of the participant (n = 99, 22%) were considered to have poor knowledge of leprosy.

Multivariate analysis showed that participants who knew someone affected by leprosy, completed higher education and health care workers all had significantly higher mean levels of knowledge of leprosy (see Table 3).

**Table 3:** Correlations between level of knowledge about leprosy and the other variables in the dataset. This model explained 16% of the variability of knowledge of leprosy (R-squared = 0.15).

				*p-*value	
		Regression coefficient	Standard error		*N*
	*Constant*	*2*.*678*	.*118*	.*000*	
	Health care worker	.912	.206	.000	50
	Completed higher education	.483	.148	.001	158
	Knows someone affected by leprosy*	.345	.134	.011	225

*) The comparison category

### Attitudes: Questions for index patients only

The final five questions of the KAP measure, about attitudes people have towards persons affected by leprosy, were asked to index patients (*n =* 100) only. Most index patients (87%) knew that leprosy can be treated in six to twelve months. Over half of the participants (56%) indicated they would prefer to keep people from knowing they have leprosy. Some participants (22%) indicated that they thought that neighbours, colleagues or others in their community have less respect for them because of their illness. A small proportion of participants (12%) said some people refuse to visit their home even after they have been treated. In addition, eight participants (8%) indicated they decided by themselves to stay away from work or a social group. It is worth noting that most participants replied that they were “not sure” about the answer to the question (ranging from 35% to 60% of the answers given).

### Attitudes, stigma and social distance

The EMIC-CSS and SDS were used to assess attitudes and stigma in contacts, community members and health care workers regarding leprosy. An overview of the scores per participant group can be found in Table 4.

**Table 4:** Mean total scores per participants group. A high score on the KAP measure reflects higher knowledge, whereas high EMIC-CSS and SDS scores reflect higher levels of stigma and desired social distance respectively.

	KAP measure (up to 17 items), range 0–8		EMIC-CSS (17-items), range 0–30		SDS (7-items), range 0–21	
	Mean (95%CI)	Range	Mean (95%CI)	Range	Mean (95%CI)	Range
Index patient	3.3 (3.08–3.52)	0–6	-	-	-	-
Close contact	3.2 (3.00–3.41)	0–5	13.9 (12.7–15.1)	0–26	7.0 (5.99–8.01)	0–21
Community member	3.0 (2.83–3.17)	0–5	16.2 (15.2–17.2)	2–30	8.2 (7.36–9.04)	0–21
Health care worker	4.2 (3.80–4.60)	0–7	14.9 (13.4–16.4)	0–24	4.2 (3.22–5.18)	0–13
All groups	3.2 (3.13–3.35)	0–7	15.3 (14.6–16.0)	0–30	7.2 (6.61–7.79)	0–21

The mean EMIC-CSS score was 15.3 (95%CI 14.6–16.0). Answers to questions that related to marriage and avoidance of persons affected by leprosy most often indicated stigma (see Figure B in S2 Fig). We found that participants who knew a person affected by leprosy had higher mean EMIC-CSS scores and therefore higher levels of perceived stigma, compared to participants who did not know a person affected by leprosy (17.3 vs 14.2, *p*<0.001, independent samples t-test). In addition, being a close contact and doing paid work were associated with lower EMIC-CSS total scores and thus lower levels of stigma (see Table 5). We found that participants who thought that leprosy is caused by an unclean environment or a divine punishment for sins, and participants who thought leprosy transmits through skin contact or by air had significantly higher mean EMIC-CSS scores (see Table 5).

**Table 5:** Correlations between level of stigma and the other variables in the dataset. This model explained 15% of the variability of stigma towards persons affected by leprosy (R-squared = 0.148).

		Regression coefficient	Standard error		N
	*Constant*	*15*.*003*	*1*.*012*	.*000*	
	Thinks leprosy transmits by air	4.461	1.531	.004	18
	Thinks leprosy is a divine punishment for sins	3.974	1.667	.018	17
	Thinks leprosy is caused by an unclean environment	2.873	1.253	.023	35
	Knows someone affected by leprosy*	-2.393	.722	.001	224
	Thinks leprosy transmits through skin contact	2.305	.731	.002	120
	Indicate they don’t know what causes leprosy	2.208	.859	.011	216
	Occupation is paid work	-1.710	.729	.020	115
	Close contact	-1.576	.760	.039	110

*) The comparison category

The mean SDS score was 7.2 (95%CI 6.6–7.8). Questions that indicated the most negative attitudes related to marriage and having someone affected by leprosy as caretaker of your children (see Figure C in S3 Fig). We found that health care workers, participants who knew someone affected by leprosy, men, and people with a higher number of correct answers on the KAP measure had lower mean SDS total scores and thus a more positive attitude (see Table 6). Community members, women and illiterate participants had higher mean SDS total scores and thus on average more negative attitudes towards persons affected by leprosy (see Table 6). In addition, participants who said they didn’t know the early symptoms of leprosy, participants who thought that leprosy is transmitted by air and participants who thought that leprosy is contagious after treatment also had more negative attitudes (see Table 6).

**Table 6:** Correlations between level of social distance and the other variables in the dataset. This model explained 19% of the variability of stigma towards persons affected by leprosy (R-squared = 0.187).

		Regression coefficient	Standard error	*p*-value	N
	*Constant*	*2*.*356*	.*949*	.*018*	
	Thinks leprosy transmits by air	3.915	1.695	.019	18
	Illiterate	2.135	.791	.011	71
	Doesn’t know the early symptoms of leprosy	2.120	.713	.002	95
	Health care worker	-2.035	.818	.013	50
	Community member	1.931	.637	.003	185
	Gender (women)*	1.722	.593	.006	122
	Thinks leprosy is contagious	.642	.319	.050	109

*) The comparison category

From the in-depth interviews it became clear that none of the six index patients who were interviewed and only one of the nine index patients who participated in the focus group discussions knew anyone else who was affected by leprosy. In addition, participants often didn’t want to disclose because of shame or to avoid negative reactions or social exclusion. One index patient explained:

*“…No*, *I did not tell my friend*. *I kept it hidden (…) because people have a bad perception about leprosy in society*. *Later people start thinking bad about it [being affected by leprosy] for instance “don't keep him with us”…” (Index patient*, *male*, *20)*

In addition, during the in-depth interviews and focus group discussions, many participants indicated that community members keep their distance from persons affected by leprosy or exclude them from social activities. Community members of participants don’t want to talk to, eat with, sit with or touch persons affected by leprosy (six out of the seven community members in the focus group discussion). Persons affected are also often not invited to ceremonies or parties. Avoiding persons affected by leprosy was often linked to the idea of transmission of leprosy by touch (14 out of the 18 non-health workers in the in-depth interviews). Over half of the participants who were interviewed (14 out of the 24 participants in the in-depth interviews) indicated that community members would refrain from touching a person affected by leprosy. Many of them indicated they also thought leprosy transmits via touch (eight participants). “Untouchability” was mentioned often. There were also participants who were positive towards persons affected by leprosy, one community member explained:

*“…Certainly it [being affected by leprosy] will not make any difference*, *everyone is given equal respect…” (Community member*, *male*, *42)*.

## Discussion

Our study revealed poor knowledge regarding leprosy among index patients, close contacts, community members and health care workers in Fatehpur district, Uttar Pradesh, India. There were few participants with adequate knowledge of leprosy, defined as 70% or more correct answers on the knowledge section of the KAP measure (≥5 out of 7 questions). Knowledge on mode of transmission, cause and symptoms of leprosy was especially poor. In addition, we found high levels of perceived stigma and desired social distance towards persons affected by leprosy.

The present study revealed that only 12.5% of the participants had adequate knowledge of leprosy, while 22% had poor knowledge. Similar findings have been reported in other studies in India. Even though other studies did not report a quantified level of knowledge, several reported that knowledge levels among persons affected and their community members were low or inadequate [24,25,27,30]. Two studies found that persons affected by leprosy had higher levels of knowledge about leprosy than community members [28] and family members [19]. This difference was also found in the present study, but was not statistically significant. In the present study, health care workers were found to have better knowledge of leprosy than other participants. This finding is similar, even though knowledge levels appear to be lower, to findings from a study that looked at knowledge, attitudes, and reported practices of health care providers regarding leprosy in Assam, northeast India. In this study over 80% of the participants had attended training programmes on leprosy [26]. In the present study this was 50%. We expect that health care workers who receive training on leprosy will have higher levels of knowledge. This assumption is supported by a study by Rao and colleagues conducted in southeast India, who found that “Medical Officers who received training in leprosy and possessed reference material on leprosy have shown higher knowledge and practice” [29].

In the present study, levels of knowledge about cause (10%), mode of transmission (5%) and early symptoms of leprosy (16%) were poor. This is much lower than other studies, who report up to 28% of correct knowledge on mode of transmission [18,27,28], 26–44% correct knowledge on cause of leprosy [12,18,20,27,28,30] and 20–73% of correct knowledge on early symptoms [12,20,24,25,28]. Only one study, among community members of urban slums in southern India, reports similar low levels of knowledge on cause, mode of transmission and early symptoms [24]. These low levels of correct knowledge may in part reflect a lack of dissemination of relevant, correct information as participants in the present study reported that their main sources of leprosy-related information were health care workers and community members. Traditional beliefs are likely to be deeply rooted in the Indian culture and can vary from state to state. We believe that traditional beliefs and a lack of knowledge of leprosy play an important role in to the persistence of stigma.

The main misconception related to cause of leprosy was that people thought leprosy is caused by an unclean environment or by a lack of hygiene. An unclean environment, the belief that leprosy is hereditary and bad blood were causes often mentioned in other studies also [12,27,28,30]. In addition, in the present study many people believed that leprosy transmits by touch. Participants reported that community members would refrain from touching a person affected by leprosy and often linked exclusion to “untouchability”. A study among persons affected by leprosy, their family members and people with non-leprosy skin diseases in a tertiary care hospital in Delhi, found something similar, stating that “fear of the leprosy-affected and reluctance for physical contact (…) were prominent” [19]. We found only one study in in Madhya Pradesh, central India, from 1981, that explicitly stated that many persons affected by leprosy (62%) experienced stigma related to touch [35].

We found that 93% of the participants knew that leprosy can be treated. Of these people, 97% knew that leprosy can be treated by medication. This is higher than in other studies in India, which reported 29 to 90% of correct knowledge about curability and treatment of leprosy [12,18,20,24,25,27,28,30].

The levels of stigma and desired social distance towards persons affected by leprosy found in the present study were high. We found that more knowledge about leprosy was associated with lower levels of stigma, but that ‘knowledge’ explained only a small proportion of the variation in stigma levels. Other studies in India also report high levels of negative attitudes and stigma [12,19,25,28,30,36–38]. A study in a tertiary care hospital in Delhi also found that greater knowledge of leprosy is a positive predictor of attitude [19]. This suggests that improving knowledge about leprosy may also improve attitudes.

In our study participants who knew a person affected by leprosy perceived higher levels of community stigma while being a close contact to someone affected was associated with lower levels of stigma. This is a surprising finding, as one would expect that close contacts of someone affected by leprosy are people who know someone affected by leprosy. Next to higher perceived levels of community stigma, participants who knew a person affected also perceived lower levels of desired social distance compared to participants who did not know a person affected. We believe that perceived stigma in the community may increase when people know someone affected and see the difficulties they experience. At the same time knowing someone affected could potentially improve personal attitudes towards the person, thus reduce the desired social distance. Furthermore, considering the high levels of incorrect knowledge of our participants regarding leprosy, we believe that the higher levels of stigma among people who know someone affected by leprosy in our study could also be due to their misconceptions regarding leprosy.

Finally, the findings of this study have to be considered in the context of its limitations. A limitation of the study is that it was a cross-sectional study and could therefore not establish definite cause-and-effect relationships; we were only able to form hypotheses about cause and effect relationships. Furthermore, although interesting and potentially relevant, it was not possible to take into account factors such as disability status and leprosy classification as we needed to focus on background characteristics which are most relevant in the context of designing large group interventions, for instance gender and level of education. In addition, the SDS used in this study had not yet been formally validated in Hindi. However, the SDS has been translated, piloted, extensively used and had its psychometric properties assessed in a parallel baseline study (Ballering, in preparation). We therefore considered the SDS a valid measure of social distance. Finally, this study only assessed leprosy-related stigma in community members, contact and health care workers and did not assess self-stigma and enacted stigma. A strength of the present study is the mixed-method approach that allowed for triangulation of the data.

The present study has important implications for the development of leprosy control strategies. This study identified a lack of knowledge about leprosy and high levels of stigma towards persons affected by leprosy in Fatehpur district, Uttar Pradesh, India. The insights we obtained in this study in knowledge gaps, beliefs and misconceptions will be used to design effective community education methods to raise awareness, positively influence the perception of and improve knowledge regarding leprosy and reduce the stigma against leprosy. We recommend a combination of written health education materials in combination with behavioural change interventions, as written materials used in isolation are often not adequate to change behaviour [39,40] and because a high level knowledge of leprosy alone does not necessarily lead to more positive attitudes towards persons affected [41]. In addition, we identified a need for increased awareness of and information about leprosy among health care workers. Even though health care workers had higher levels of knowledge than the other participants in this study, they were still not adequate. We recommend standard training on leprosy for all health care workers and regular refresher courses in areas that are endemic for leprosy. We expect that these education methods will improve strategies for early case detection in leprosy thus improving the effectiveness of the National Leprosy Eradication Programme.

### Conclusion

This study revealed poor knowledge regarding leprosy among index patients, close contacts, community members and health care workers in Fatehpur district, northern India. Knowledge on mode of transmission, cause and symptoms of leprosy was especially poor. In addition, we found high levels of stigma towards persons affected by leprosy.

Several factors were associated with higher levels of negative attitudes towards persons affected by leprosy, including knowing a person affected by leprosy, being a community member, being a woman, being illiterate and specific conceptions regarding cause, transmission and contagiousness of the disease. We found that better knowledge of leprosy was associated with lower levels of social distance towards persons affected by leprosy.

In order to improve knowledge, reduce misconceptions and positively influence the perception of leprosy, community education is needed. Special emphasis needs to be placed on education regarding mode of transmission, cause and symptoms of leprosy. A multidisciplinary approach that takes cultural beliefs, knowledge gaps and fears into consideration is recommended.

## Supporting information

S1 Checklist STROBE Checklist. (DOC)

S1 Fig The percentage of correct answers per KAP knowledge question (in the presence of incorrect answers; n = 446). Legend: the dark green bars indicate the number of participants who gave the correct answer to the question, the light green bars the number of participants who gave the correct answer in the presence of incorrect answers and the red bars the number of participants who answered correctly or said they didn’t know.
(TIF)

S2 Fig Reponses per EMIC-CSS stigma scale question. **Responses from close contacts (n = 111), community members (n = 185) and health care workers (n = 50).** Legend: the red bars indicate the number of participants who replied “yes”, the yellow bars “possibly” and the green bar “no” or “don’t know”.
(TIF)

S3 Fig Reponses per SDS question. **Responses from close contacts (n = 111), community members (n = 185) and health care workers (n = 50).** Legend: the red bars indicate the number of participants who replied “definitely not willing”, the orange bars “probably not willing”, the light green bars “probably willing” and the dark green bars “definitely willing”.
(TIF)
