# Correction: The role of perceptions and knowledge of leprosy in the elimination of leprosy: A baseline study in Fatehpur district, northern India

**DOI:** 10.1371/journal.pntd.0010519

**Published:** 2022-06-06

**Authors:** Anna T. van ‘t Notableordende, Ida J. Korfage, Suchitra Lisam, Mohammed A. Arif, Anil Kumar, Wim H. van Brakel

There is an error in the fifth sentence in the Results sub-section Attitude, stigma and social distance. The correct sentence is: We found that participants who knew a person affected by leprosy had lower mean EMIC-CSS scores and therefore lower levels of perceived stigma, compared to participants who did not know a person affected by leprosy (14.2 vs 17.3, *p*<0.001, independent samples t-test).

The seventh paragraph in the Discussion section misreports the study findings. The paragraph should read: In our study participants who knew a person affected by leprosy and those who were a close contact of someone affected perceived lower levels of stigma. Knowing a person affected also appeared to reduce the desire for social distance towards leprosy patients, but this effect did not quite reach statistical significance in the multivariate analysis. Nevertheless, it seems that knowing someone affected could potentially improve personal attitudes towards and reduce fear of person affected.

There is an error in the first column heading in Table 4. Please see the correct Table 4 below.

In Tables 3 and 5, there is an incidental asterisk footnote that should not be present. Please see the correct Tables 3 and 5 below.

**Table 3:** Correlations between level of knowledge about leprosy and the other variables in the dataset. This model explained 16% of the variability of knowledge of leprosy (R-squared = 0.15).

		Regression coefficient	Standard error	*p-*value	N
	*Constant*	*2*.*678*	.*118*	.*000*	
	Health care worker	.912	.206	.000	50
	Completed higher education	.483	.148	.001	158
	Knows someone affected by leprosy	.345	.134	.011	225

**Table 4:** Mean total scores per participants group. A high score on the KAP measure reflects higher knowledge, whereas high EMIC-CSS and SDS scores reflect higher levels of stigma and desired social distance respectively.

	KAP measure (7 knowledge items), range 0–7		EMIC-CSS (17-items), range 0–30		SDS (7-items), range 0–21	
	Mean (95%CI)	Range	Mean (95%CI)	Range	Mean (95%CI)	Range
Index patient	3.3 (3.08–3.52)	0–6	-	-	-	-
Close contact	3.2 (3.00–3.41)	0–5	13.9 (12.7–15.1)	0–26	7.0 (5.99–8.01)	0–21
Community member	3.0 (2.83–3.17)	0–5	16.2 (15.2–17.2)	2–30	8.2 (7.36–9.04)	0–21
Health care worker	4.2 (3.80–4.60)	0–7	14.9 (13.4–16.4)	0–24	4.2 (3.22–5.18)	0–13
All groups	3.2 (3.13–3.35)	0–7	15.3 (14.6–16.0)	0–30	7.2 (6.61–7.79)	0–21

**Table 5:** Correlations between level of stigma and the other variables in the dataset. This model explained 15% of the variability of stigma towards persons affected by leprosy (R-squared = 0.148).

		Regression coefficient	Standard error	*p*-value	N
	*Constant*	*15*.*003*	*1*.*012*	.*000*	
	Thinks leprosy transmits by air	4.461	1.531	.004	18
	Thinks leprosy is a divine punishment for sins	3.974	1.667	.018	17
	Thinks leprosy is caused by an unclean environment	2.873	1.253	.023	35
	Knows someone affected by leprosy	-2.393	.722	.001	224
	Thinks leprosy transmits through skin contact	2.305	.731	.002	120
	Indicate they don’t know what causes leprosy	2.208	.859	.011	216
	Occupation is paid work	-1.710	.729	.020	115
	Close contact	-1.576	.760	.039	110
