# Supplementary figures and images for: The role of perceptions and knowledge of leprosy in the elimination of leprosy: A baseline study in Fatehpur district, northern India
Source: PLoS Negl Trop Dis. 2019 Apr 5;13(4):e0007302. doi: 10.1371/journal.pntd.0007302 (PMC6469810; doi:10.1371/journal.pntd.0007302)

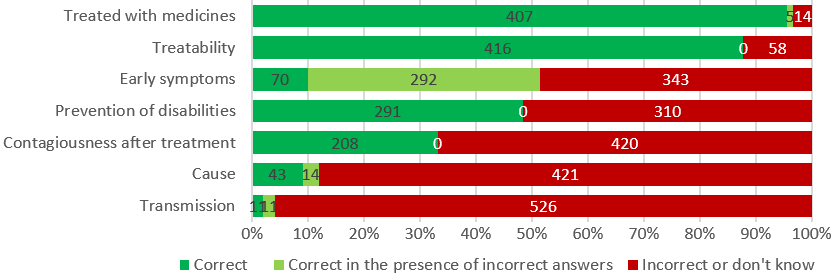

Supplement: S1 Fig — Legend: the dark green bars indicate the number of participants who gave the correct answer to the question, the light green bars the number of participants who gave the correct answer in the presence of incorrect answers and the red bars the number of participants who answered correctly or said they didn’t know. (TIF) [file pntd.0007302.s002.tif]

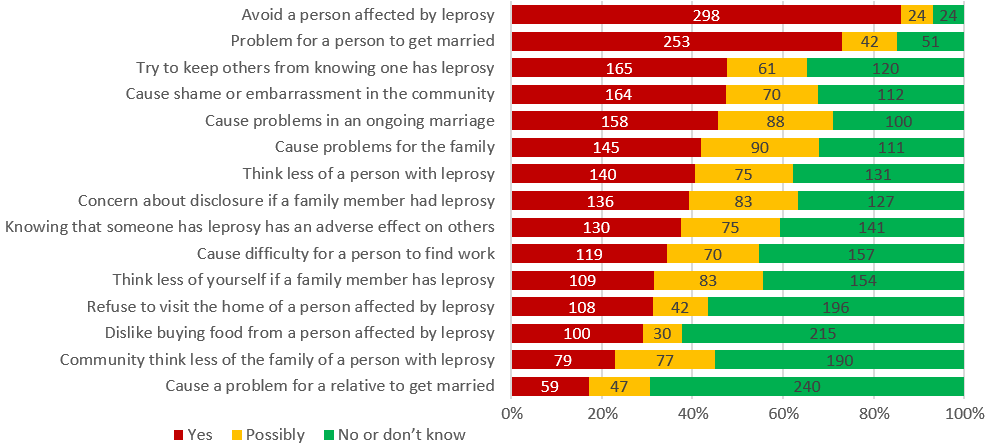

Supplement: S2 Fig — Responses from close contacts (n = 111), community members (n = 185) and health care workers (n = 50). Legend: the red bars indicate the number of participants who replied “yes”, the yellow bars “possibly” and the green bar “no” or “don’t know”. (TIF) [file pntd.0007302.s003.tif]

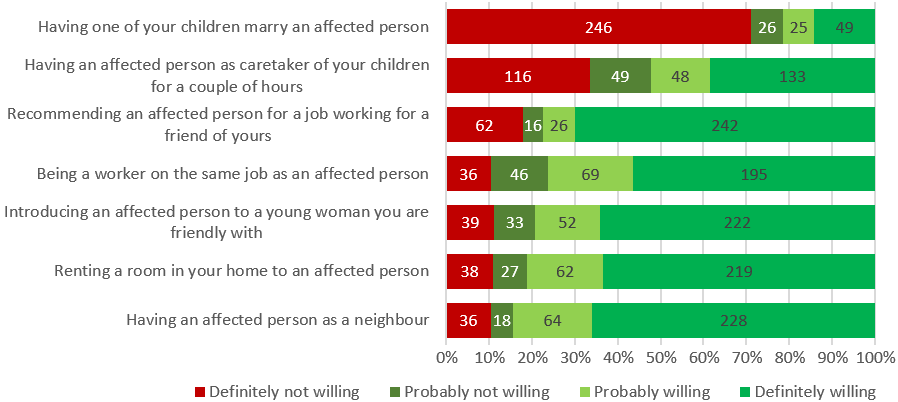

Supplement: S3 Fig — Responses from close contacts (n = 111), community members (n = 185) and health care workers (n = 50). Legend: the red bars indicate the number of participants who replied “definitely not willing”, the orange bars “probably not willing”, the light green bars “probably willing” and the dark green bars “definitely willing”. (TIF) [file pntd.0007302.s004.tif]
